# Graph Constrained Discriminant Analysis: A New Method for the Integration of a Graph into a Classification Process

**DOI:** 10.1371/journal.pone.0026146

**Published:** 2011-10-14

**Authors:** Vincent Guillemot, Arthur Tenenhaus, Laurent Le Brusquet, Vincent Frouin

**Affiliations:** 1 Laboratory of Functional Genomics – CEA, DSV, IRCM, Evry, France; 2 Department of Signals and Electronics Systems – Supélec, Gif-sur-Yvette, France; University of Minnesota, United States of America

## Abstract

Integrating gene regulatory networks (GRNs) into the classification process of DNA microarrays is an important issue in bioinformatics, both because this information has a true biological interest and because it helps in the interpretation of the final classifier. We present a method called graph-constrained discriminant analysis (gCDA), which aims to integrate the information contained in one or several GRNs into a classification procedure. We show that when the integrated graph includes erroneous information, gCDA's performance is only slightly worse, thus showing robustness to misspecifications in the given GRNs. The gCDA framework also allows the classification process to take into account as many *a priori* graphs as there are classes in the dataset. The gCDA procedure was applied to simulated data and to three publicly available microarray datasets. gCDA shows very interesting performance when compared to state-of-the-art classification methods. The software package gcda, along with the real datasets that were used in this study, are available online: http://biodev.cea.fr/gcda/.

## Introduction

Very often, biologists and bioinformaticians have prior knowledge about the relationships that exist between genes under specific biological conditions. These structured priors are usually represented by a graph, called a gene regulation network (GRN) throughout this paper, in which the nodes are the genes and the edges represent interactions between genes. Integrating such a structured prior knowledge into the classification of microarray data is an important bioinformatics research field and has recently been addressed in the literature (for example, [1]–[4]). The properties of the Laplacian's graph eigen values are used by Rapaport *et al.* [2] and Li *et al.* [1] to compute a classifier intended to be “smooth” across the graph. Zhu *et al.* [3] encodes the graph by means of additional specific constraints in the support vector machines (SVM) [5] optimization problem (in a way that is also suggested by Rapaport *et al.* [6]). Binder *et al.* [4] proposed that the graph be incorporated in a boosting framework. All of these methods pursue the same general objective: two variables connected in the GRN must have close weights in the classification function. This type of constraint yields a better interpretability of the resulting classifier, but not necessarily better performance.

In this paper, we propose a new method - called graph constrained discriminant analysis (gCDA) - , which is a constrained version of the discriminant analysis [7], with constraint depending on information that is represented by one or more graphs. Here, we present a fully operational and validated method that has resulted from preliminary works reported in [8]. In the discriminant analysis (DA), the decision function involves the inverse of the within-class covariance matrix. In the high-dimensional setting (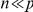) considered here, the usual maximum likelihood covariance estimator is singular. As a result, the use of shrinkage estimators for the covariance matrix is needed, as described in the regularized discriminant analysis (RDA) [9]. Our approach is two-fold: first, the within-class covariance estimation is shrunk by integrating the information contained in GRNs. Then, the new estimator is entered into a DA framework. The underlying motivation for this approach is to improve the accuracy of the predictions, at least when compared to RDA.

The present work is structured as follows: the first section is dedicated to the presentation of gCDA and of the state-of-the-art methods to which it is compared. The second part is devoted to the validation of gCDA on a simulated dataset and three publicly available gene expression microarray datasets [10]–[12].

## Methods

The integration of a graph into the classification process of microarray data requires that GRNs describing the dependencies between genes and a given microarray dataset are considered. The structure of these two objects is radically different, and the challenging task we attempt to overcome in this paper is to combine these two sources of information.

### Notations

Let 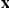 be a 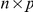 matrix containing the expression profiles of the 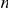 individuals distributed in two classes. Each individual is associated with a response variable. In this paper, we evaluate only binary classification problems: an individual from class 1 will have a response variable equal to 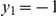 and equal to 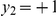 otherwise. The set of the individuals belonging to class 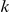 is denoted by 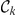. Let 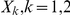 be the two 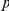-variate random variables that model the expression of the 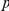 genes in each class. The means and covariance matrices of these variables will be denoted as 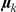 and 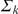, respectively. Moreover, the variables 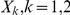 are supposed to be multivariate and following Gaussian distributions. Therefore, an individual from the dataset, 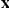, is a realization of a multivariate Gaussian mixture variable of density 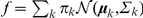, with 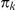 representing the probability that an individual belongs to class 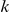.

We consider finite, undirected graphs to model GRNs. A graph, 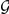, is an object defined by the set of its edges, 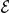, and the set of its vertices, 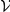. A vertex represents a gene. Hence, 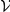 contains 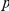 vertices. Let 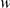 be the function 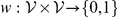 such that 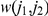 is 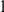 if there is an edge between vertices 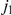 and 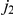 and 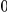 otherwise.

The Laplacian of a graph 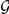, denoted by 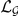, is a semi-definite, positive, 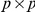 matrix whose coefficients are:
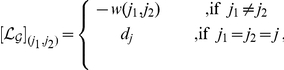



with 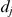 representing the connectivity degree of vertex 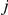. Thus, each null term in 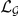 corresponds to an absence of an edge in 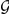.

### Related work

Rapaport *et al.* [2] proposed that a spectral transformation be applied to the Laplacian 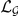. This gives a semi-definite, positive matrix, which is then used as a kernel matrix that is loaded into SVM. The authors of this work do not report any improvement of the performance of classification, but they suggest that this approach results in better interpretability of the classification model.

In a more recent study [6], Rapaport *et al.* integrate the given graph by adding constraints to the classical SVM optimization problem. These additional constraints encode the fact that two adjacent variables must have close weights in the final model. This idea is further developed by Zhu *et al.* [3], who proposed a method called network-based (NB)-SVM. This approach aims to solve the following optimization problem:
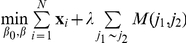


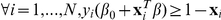


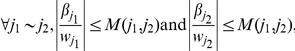



with 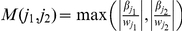, where 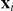 the expression profile of the 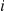
th individual and 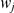 a weight that is dependent on the degree 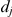 of gene 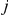 in the graph 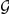. The values proposed by [3] are 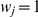, 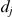 or 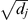. In the comparison presented in our paper, we considered the case 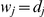.

The two methods described above are intended to solve the issue that variables connected in a given graph must have close coefficients in the decision function. This type of constraint clearly helps the interpretation of the resulting classifier, but it is not specifically designed to improve the performance of the classification, even if Zhu *et al.* [3] show results on simulated data that support such an improvement. By contrast, the method proposed here is explicitly designed to improve the classification accuracy.

In a nutshell, we propose to regularize the estimation of the covariance matrix by integrating information contained in the GRN(s). The resulting estimator can then simply be used in the context of DA. As described by [2], the key element of our integration procedure is the 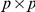 Laplacian matrix of 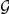. As we will see in the section describing the Gaussian graphical model, the Laplacian matrix can be considered to be homogeneous to the inverse of a covariance matrix and will be used in our shrinkage target.

### Discriminant Analysis

DA is a simple, yet very popular, classification method [7], [13]. To implement gCDA, we focused more particularly on the Fisher's DA. This analysis aims to first determine a linear transformation, defined as 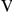, of the dataset that is able to maximize the between versus within-class covariance ratio:
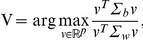



with 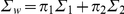, the within-class covariance matrix and 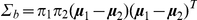, and the between-class covariance matrix.

Considering that there are only two classes, the transformation 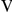 defines a 1-dimensional space: the discriminant axis. Once an individual 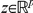 is projected onto this discriminant axis, one can predict its class based on the following Bayesian decision function:
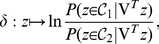
(1)if 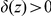, it is decided that 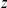 belongs to class 1. Otherwise, 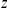 is attributed to class 2. The Gaussian assumption helps substantially to simplify the expression of 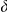 because 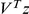 is the realization of either a Gaussian variable 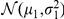 or 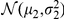, with probabilities equal to 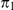 and 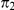, respectively. This formula (1) can be rewritten as:
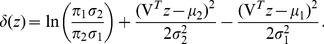
(2)


It was shown (for example, [14]) that the unknown parameters of 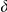, defined in the equation (2), can be re-expressed as a function of 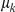, 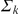:
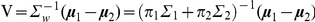


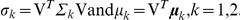



Moreover, we can consider the linear and quadratic cases in the DA framework:

• in the linear case, 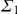 is supposed to be equal to 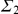. If this is the case, then 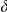 is a linear function of the components of 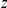.

• in the quadratic case, 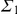 and 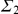 are supposed to be different from each other, and, as a result, 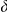 is a quadratic function of the components of 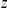. Moreover, the quadratic case allows us to consider situations in which the GRNs from the two classes are different. In that case, we can integrate one GRN per class. Such an interesting property cannot be found in the methods presented in the literature [2],[1],[3], although it could be of interest to the biologist. Indeed, for example, new approaches have been developed with the purpose of estimating differences between GRNs that exist between two classes of patients [15]. Plus, the fact that our method is able to integrate one GRN per class is of interest in the cases where unexpected differences in phenotype are observed (e.g. some types of cancer with similar excision histology but different survival times). In those cases, differences in the connectivity of the GRN might be expected and may help in building a two-class predictor. Conversely, a better classification rate obtained when using two GRN variants could bring a validation of their biological relevance.

Due to the 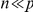 setting, the estimation of the covariance matrix used in the discriminant analysis has to be regularized. In gCDA, the GRNs are integrated into the covariance matrix estimator using Gaussian graphical models, hence realizing at the same time the needed regularization.

### Gaussian Graphical Models

The theory of Gaussian graphical models [16] (GGM) allows for the description of the dependencies between variables by a graph and the formulation of correspondences between the graph and the covariance matrix of the considered Gaussian variables. Let 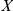 be a random, multivariate, Gaussian variable with mean 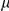 and covariance matrix 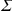. According to GGM, two variables, 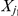 and 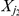, are independent conditionally to the remaining variables if 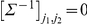. If the graph 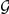 describes the conditional independence between variables, then 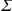 has to respect the constraint:
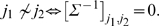
(3)


With this property in mind, we propose the following shrinkage target, which integrates the *a priori* information encoded in 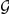:
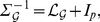
(4)where 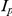 is the 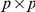 identity matrix and the Laplacian matrix 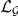 is a semi-positive matrix respecting (3).

### Integrating the GRN

In the 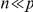 case, the empirical covariance matrix, 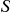, is an unbiased estimator of the covariance matrix, but it shows poor performance with regard to its variance.

Guo *et al.* [17] propose to regularize this estimator in the following way: 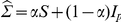. Schäfer *et al.* [18], propose to replace 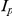 with the so-called “target matrix” and provide a closed-form expression for the parameter 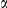. Our method is inspired by those ideas: in gCDA, we use the model (4) to build our own target matrix 
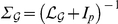
, which in turn is used to regularize the estimation of the covariance matrix
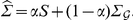



The value of the parameter 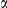 is determined with a cross-validation procedure. Let us note thereafter 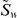 the estimation of the within-class covariance matrix we propose.

In the Linear gCDA, each class is supposed to have the same covariance, and there is only one GRN:
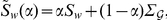



with 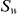 representing the empirical, within-class covariance matrix.

In the Quadratic gCDA, each class is characterized by a different GRN:
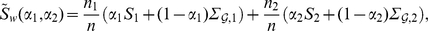



with 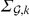 and 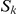 representing the target matrix and the empirical covariance matrix for class 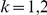, respectively. The quadratic gCDA allows for the integration of two graphs, corresponding to two biological situations, into a classification process.

## Results

In this section, we apply gCDA to simulated and real datasets. The performance is evaluated in a Monte Carlo cross validation (MCCV) framework: The dataset is randomly split into a training dataset (two thirds), and the rest of the dataset is used as a test dataset. The whole procedure is iterated 100 times. The tuning parameters (e.g. 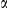 or 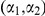 for gCDA) of the considered classification methods are computed with an internal, 10-fold cross validation. We compared gCDA to the network-based support vector machines (NB-SVM) presented by Zhu *et al.* and to the reference method they considered [3], namely linear programming (LP)-SVM [19]. Rapaport's method is not considered in the comparison because the authors stated that it does not perform better than a regular SVM classification. We also computed the performance of the regular SVM method (as implemented in the R package e1071 [20]) and the RDA (implemented in the package rda [21]).

The results presented here were obtained from simulated and microarray data. In the latter case, the performance assessment and comparisons were performed while varying the method to get the GRN and the number of GRN nodes.

### Results obtained on simulated datasets

**Figure 1:**
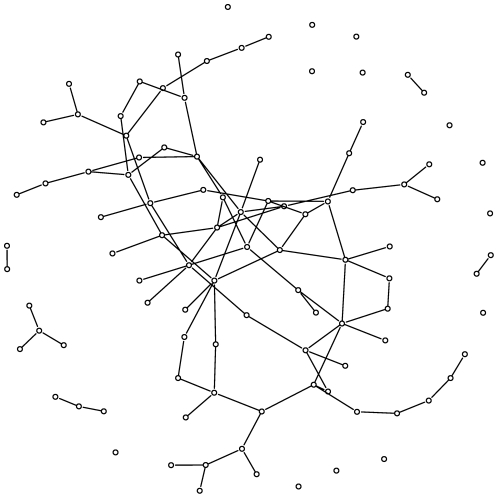
Graph used to generate simulated data: an Erdös-Rényi graph.

To demonstrate the performance of the presented methods, we generated simulated data. We used Erdös-Rényi's graphs (see Figure 1) to model the interactions between genes, which allows loops, hubs, and multiple connected components. We used the following algorithm:

- compute one Erdös-Rényi graph 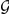 for both classes or two graphs 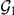 and 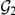 (one per class),
- for each graph, use the model given in equation (4) to build a covariance matrix,
- and model the two classes by random multivariate Gaussian variables 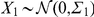 and 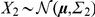. 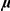 represents the mean difference between the two datasets.

A comparison of the results obtained from simulated data for gCDA, NB-SVM, RDA, and SVM is presented in Table 1. The key result of these simulations is that the integration of the known model for the covariance matrix greatly improves the classification performance (see the performance of RDA compared to the results of gCDA). Finally, the performance is always better for gCDA than NB-SVM, the other method that integrates the known graph.

**Table 1:** Results using simulated datasets.

Setting	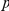	RDA	SVM	LP-SVM	NB-SVM	gCDA
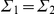	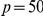	66.12 (13.79)	80.32 (6.55)	69.97 (10.04)	70.24 (10.54)	88.74 (5.07)
	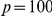	76.00 (21.37)	92.59 (3.58)	70.91 (11.90)	74.76 (9.70)	96.56 (2.81)
	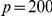	65.26 (19.36)	81.24 (7.21)	70.56 (13.10)	67.06 (8.79)	93.38 (4.13)
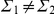	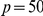	71.44 (12.90)	77.50 (6.43)	71.97 (9.09)	70.94 (9.06)	80.29 (6.24)
	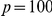	70.59 (18.73)	84.47 (5.76)	71.59 (9.97)	70.47 (9.79)	86.65 (5.92)
	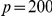	72.35 (21.70)	87.50 (5.44)	73.65 (12.57)	73.74 (11.77)	92.56 (4.66)

Mean of the good classification percentage (and standard deviation) over 100 MCCV iterations. Results obtained using simulated datasets. 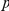 is the number of variables. The number of individuals is set to 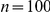. We used the linear version of gCDA when 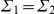 and the quadratic version when 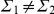.

To explore the limits of gCDA, we also run the linear version of the method on a simulated dataset containing 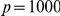 variables and 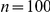 individuals split into two classes. The computation times of a single MCCV iteration lasted 667.82 s for gCDA against 12.65 s for SVM (on a personal computer with a processor Pentium(R) Dual core CPU E5800 3,20GHz×2 and 3.42 GB RAM). It has to be stated that the methods LP-SVM and NB-SVM could not be used on this dataset due to limited computer memory. The results are quite interesting, since SVM (86% of mean good classification rate) performs as well as gCDA (87%), whereas RDA performs as bad as a random assignment of the classes (47%). It shows that the regularization of the estimation of covariance matrices we apply in gCDA is more efficient than the one in RDA.

**Figure 2:**
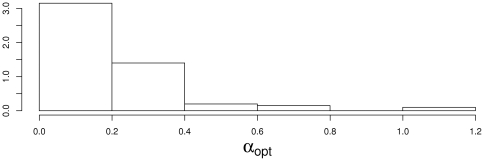
Histogram of the optimal values of 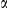. These values were selected by 10-fold cross validation obtained on simulated data (linear setting, 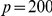 and 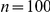).

Additionally, Figure 2 depicts the values of the parameter 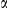 that was selected by cross validation: the selected values are close to 0, which reveals that the graph was taken into account in the estimation of the covariance matrix.

The classification performance of gCDA also depends on the quality of the integrated graph.

**Figure 3:**
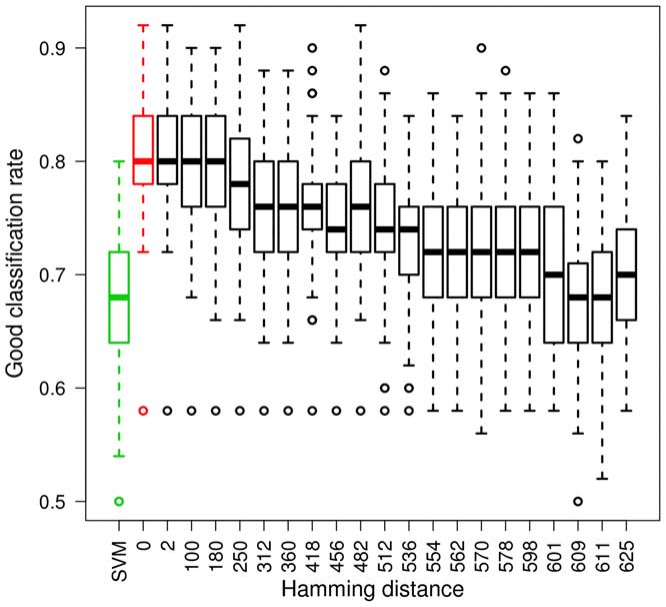
Plot of the classification performance as a function of the Hamming distance between the real graph and the graph integrated in gCDA. For this part of the simulation study, the number of variables is set to 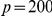 and the number of individuals to 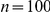.

We empirically observed the evolution of gCDA's performance as a function of the quality of the integrated graph on simulated data. Starting with the graph that was used to simulate the dataset, we generated a set of gradually different graphs by randomly reassigning some of its edges to different vertices. The difference between two graphs is calculated as the number of different vertices between the union of the two graphs and their intersection, which corresponds to the structural Hamming distance [22] because the considered graphs are undirected. The results are shown on Figure 3. Although the best results are obtained with the real graph, our method performs robustly in spite of misspecified edges in the integrated graph. Moreover, we see that gCDA maintains its performance at least at the same level as the SVM's, even when the graph is incorrectly specified.

### Results obtained from gene expression microarray datasets

To evaluate the performance of gCDA with real data, we chose three gene expression microarray datasets. The characteristics of the three datasets are summarized in Table 2. These datasets are available from the Gene Expression Omnibus (GEO) public database [23] and pertain to colon [10], prostate [11] and lung [12] cancers.

**Table 2:** Characteristics of the datasets.

outcome	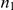 : 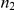	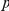	Disease	Reference	Network inferred on
control/tumor	30∶12	97	colon cancer	[10]	The rest of the original dataset
control/tumor	50∶52	282	prostate cancer	[11]	Another dataset [31]
relapse/no relapse	69∶69	325	lung cancer	[12]	Another dataset (GSE8332)

Summary of the characteristics of each of the datasets. 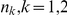 represents the number of individuals in the class, 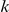. The last column indicates whether the networks are inferred on an independent part of the dataset or on another dataset. In both cases, the dataset used to compute the networks is never used in the classification process.

When dealing with gene expression microarray datasets obtained from specific tissues and under particular experimental conditions with the gCDA method, two major issues must be pointed out: 1) the graph describing the various interactions between genes is not known and has to be inferred, and 2) differences between the covariance matrices of the two classes should be evaluated. In the results reported for the real data, we investigated pragmatic choices. We selected two recognized approaches to infer the GRNs to be integrated in the classification and we also built GRNs based on reported interactions gathered in the Kyoto Encyclopedia of Genes and Genomes (KEGG) [24] database. Namely, the three sources we used to get GRNs are

- ARACNE [25], a method based on the computation of mutual information, implemented in the package minet [26],
- ridge.net [27], based on the estimation of the partial correlation matrix, implemented in the package parcor [28],
- and the KEGG pathway hsa05200, that pertains to the biological samples we considered. The network was extracted thanks to the Bioconductor library KEGGgraph [29].

For the two first kinds of GRN mentioned above, we inferred the graphs based on a dataset independent from the one used for the classification process.

These methods impose limits on the set of variables to be considered down to several hundreds. Therefore, we selected a restricted set of genes corresponding to the KEGG pathway for human cancer (hsa05200). To avoid any bias in the classification process, we never used the same dataset to compute the GRNs and to measure the classification performance. We considered a couple of distinct datasets (see Table 2) corresponding to the same tissue and pathology: one dataset was used to infer the graphs and the other was used to build the classification models. To test the hypothesis that several covariance matrices are different, we used a statistical test adapted to high dimensional datasets presented in [30]. We used simulated data to ensure that this test is, indeed, able to distinguish between situations where the covariance matrices are equal or different. The results are not shown, but the reader is encouraged to run the example implemented in the package gcda. This test was applied to the three datasets; the obtained p-values are summarized in Table 3. It appears that the quadratic version of gCDA has to be applied only to the dataset on prostate cancer.

**Table 3:** Test on the covariance matrices.

	Colon	Lung	Prostate
p-value	0.26	0.65	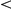 1e-3

We tested each dataset to determine whether the covariance matrices are statistically similar. The test we chose is robust enough to handle instances in which the number of variables is of the same order as the number of individuals. The null hypothesis is “
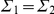
”. As a result, we rejected the null hypothesis when the p-value was lower than the threshold of 0.05.

When necessary, we re-annotated the probe sets to associate them with a corresponding specific gene (essentially for Affymetrix chips). We used the UCSC database (http://genome.ucsc.edu/, March, 2006 (NCBI36/hg18)). For each gene, we chose the probe set whose position is the closest to the transcription initiation site. When several probe sets were selected, the mean value of the measurements was computed.

As shown in Table 4, gCDA's performance - when coupled with GRNs inferred with ARACNE - was always at least as good as the performance of SVM with a linear kernel. More importantly, gCDA always outperformed NB-SVM and RDA. The fact that gCDA outperforms RDA is indeed very interesting: it shows that the method we propose to regularize the estimation of the covariance matrix is efficient even on real datasets, when the real network is not known.

**Table 4:** Comparison of gCDA's performance with the performance of three other classification methods.

	gCDA	RDA	SVM	NB-SVM
Colon	79.36 (9.63)	69.50 (13.62)	75.07 (9.87)	54.57 (22.83)
Lung	55.93 (6.00)	49.13 (6.68)	55.02 (6.12)	50.41 (6.09)
Prostate	87.10 (5.59)	64.88 (12.1)	88.62 (5.38)	56.12 (13.2)

Comparison of the performance of gCDA with the performance obtained with RDA, SVM and NB-SVM. For NB-SVM and gCDA, we chose to integrate the GRNs inferred with ARACNE. In this table are presented the mean (standard deviation) of the good classification rate over 100 MCCV iterations.

More importantly, we also assessed the way gCDA depends on the information in the GRNs by integrating three different types of GRNs: GRNs inferred with ridge.net or ARACNE and GRNs extracted from the KEGG database. The comparison between the obtained performance is presented on Table 5. This table shows that the nature of the network integrated into the classification thanks to gCDA has an interesting influence on the classification performance. For the three real datasets we analyzed, the performance yielded with the KEGG network matches the performance obtained with one of the inferred networks for the colon cancer dataset and the lung cancer dataset, but the top performance is always achieved when using an inferred metwork. It is not such an unexpected result, since KEGG reports multiple types of gene interactions which are not necessarily relevant for transcriptomic data. On the contrary, when inferring a graph with ridge.net or ARACNE, the interactions are estimated directly on gene expression datasets, hence resulting into graphs that are much more in adequacy with the data.

**Table 5:** Performance of the considered classification methods on three gene expression microarray datasets.

	ridge.net	ARACNE	KEGG
Colon	67.857 (11.77)	70.357 (11.37)	66.143 (12.17)
Lung	59.413 (5.88)	56.457 (6.31)	56.37 (5.83)
Prostate	87.441 (6.09)	87.029 (5.40)	84.353 (6.78)

The graphs integrated in the classification methods NB-SVM and gCDA were either inferred with two methods, ridge.net and ARACNE, or extracted from KEGG. In this table are presented the mean (standard deviation) of the good classification rate over 100 MCCV iterations.

Taken together, the results given in Table 4 and Table 5 show that gCDA integrates successfully GRNs into the building of the classification function. It appears to be robust enough to compensate for errors in the graph. Those results also illustrate the interest of choosing pertinent GRNs. Due to the integration of the KEGG pathways, some variables had to be removed from the analysis, which explains the differences between the two tables for gCDA (ARACNE). In addition to these results, Table 6 presents the edge differences between the three integrated GRNs for each dataset.

**Table 6:** Comparison of the integrated graphs.

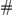 edges in:	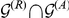	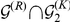	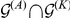	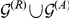	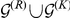	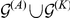
Colon	35	2	6	315	158	344
Lung	263	62	18	3204	3311	1680
Prostate	69	4	19	1099	1300	1979

Comparison of the structure of the integrated graphs using ridge.net (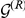), ARACNE (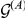) or KEGG (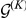). The table contains the number of edges in the intersection and the union. When two graphs were inferred, they were simply merged into a unique graph.

To conclude this section, we report the results obtained by applying gCDA to real microarray datasets while considering more than 1000 genes. The R Bioconductor package KEGGgraph was used to extract the extended version of the hsa05200 pathway from KEGG. This pathway was integrated into the classification process of lung and prostate cancer datasets. The colon cancer dataset was not considered in this part of the analysis, since only approximately 200 of its collection's genes belonged to the extended KEGG pathway. For the lung cancer dataset, there were 1252 genes in common between the collection probe sets and the extended KEGG pathway. For the prostate cancer dataset, there were 1033 genes in common between the collection probe sets and the extended KEGG pathway. We then applied linear gCDA on these two real datasets. Table 7 shows the results of these two experiments in terms of the mean of good classification rate over 100 MCCV iterations. The results obtained in this high dimensional setting raise two remarks. First, it is worth mentioning that the comparison of the mean performance is always favorable to gCDA. However, given the standard deviation, the difference between gCDA and SVM performances may not be significant. Second, the comparison between RDA and gCDA is remarkable. Indeed, the sole difference between these two methods is that for gCDA the within-class covariance estimation is shrunk by integrating KEGG prior information. From Table 7, we can observe a stable and significant improvement produced by the incorporation of the KEGG pathway information.

**Table 7:** Linear gCDA applied on high dimensional microarray datasets.

	SVM	RDA	linear gCDA
Lung	59.74 (6.65)	49.30 (6.93)	60.44 (7.32)
Prostate	84.68 (5.69)	71.59 (10.35)	85.06 (5.81)

Application of gCDA to more than 1000 variables. Comparison of SVM, RDA and linear gCDA on the lung and prostate cancer datasets: mean (standard deviation) of good classification rate over 100 MCCV iterations.

## Discussion

### Performance of gCDA

In this work, we propose a binary supervised classification algorithm of gene expression datasets that is able to integrate the information contained in gene regulation networks. The performance of gCDA is always equal to, or better than, classical SVM. When compared to state-of-the-art methods that integrate a graph, we show a significant improvement in classification performance. This result holds true whether the underlying graph is known, in the case of simulated data, or when the underlying graph of regulation is inferred, in the case of real microarray data. On real datasets, however, our method seems not to clearly outperform SVM. However, the increase in performance from RDA to gCDA, both methods based on discriminant analysis, shows that the regularization of the covariance matrix we propose is promising.

### Choice of the graph integrated in gCDA

The pipeline proposed in this paper consists of two parts, the graph inference, based on classical methods, and a second step that relies on an original constrained classification algorithm. These two parts raise two major issues. First, it must be noticed that the graph describing the various interactions between genes is not known. It has to be inferred from another dataset or from a graph that has been extracted from referenced interactions available on public databases. Second, it is usually not clear whether the covariance matrices of the two classes are different or the same.

These issues have not been addressed very often in the literature. In the procedure proposed by Rapaport *et al.*, Zhu *et al.*, Li *et al.* and Binder *et al.* [1]–[4] general GRNs are extracted from public knowledge databases, such as KEGG and subsequently integrated into the classification process. This kind of GRNs describes very general interactions between genes (like promoter-regulee or protein-protein interactions) and their adequacy to the biological process under study is difficult to assess without a thorough study by a specialist. We showed on real datasets that when a GRN extracted from a public database (KEGG) is used within gCDA, the resulting classification is worse than when inferred networks are used. We exemplified that one has to be very cautious when choosing a GRN to integrate into the classification process.

### Linear *vs* Quadratic gCDA

To determine whether the covariance matrices of the two classes are different, we propose to use a statistical test adapted to high dimensional datasets presented in [30]. The result of this test allows choosing between the linear or the quadratic version of gCDA. The fact that it allows to integrate one GRN per class if needed is a unique feature of our method compared to other classifiers.

### Impact of GRN integration

In our comparison, we restricted the reference methods to those with a direct connection to the NB-SVM method (LP-SVM and SVM) and to gCDA (RDA) to focus on how much the integrated graph can improve the classification performance. Both the interpretability and the performance of our classifier is clearly not necessarily improved compared to the approach of Rapaport *et al.*, for example, probably because of the complexity of the automatically inferred network. Apart from the fact that there may be incorrect edges, another important feature of real networks is that the weight associated to each edge is also unknown. gCDA copes with this issue by assuming an arbitrary model between the network structure and the weights. This characteristic may explain why there is no definitely significant improvement over SVM in our applications on real datasets. Future work will be dedicated to the estimation of these weights. Nevertheless, our method still shows promising classification performance on both simulated and real datasets with various complexities.
